# Formation of Polyploid Giant Cancer Cells Involves in the Prognostic Value of Neoadjuvant Chemoradiation in Locally Advanced Rectal Cancer

**DOI:** 10.1155/2019/2316436

**Published:** 2019-08-29

**Authors:** Fei Fei, Mingqing Zhang, Bo Li, Lizhong Zhao, Hui Wang, Lina Liu, Yuwei Li, Po Ding, Yanjun Gu, Xipeng Zhang, Tao Jiang, Siwei Zhu, Shiwu Zhang

**Affiliations:** ^1^Department of Pathology, Tianjin Union Medical Center, Tianjin 300121, China; ^2^Nankai University School of Medicine, Nankai University, Tianjin 300071, China; ^3^Department of Colorectal Surgery, Tianjin Union Medical Center, Tianjin 300121, China; ^4^Departments of Radiology, Tianjin Union Medical Center, Tianjin, China; ^5^Department of Epidemiology, Tianjin Colorectal and Anal Disease Research Institute, Tianjin, China; ^6^Departments of Emergency, Tianjin Union Medical Center, Tianjin, China; ^7^Department of Pathology, Affiliated Hospital of Logistic University of People's Armed Police Force, Tianjin 300071, China; ^8^Department of General Surgery, Tianjin Union Medical Center, Tianjin 300121, China; ^9^Tianjin Union Medical Center, Tianjin 300121, China

## Abstract

We previously reported that polyploid giant cancer cells (PGCCs) exhibit cancer stem cell properties and can generate daughter cells with the epithelial-mesenchymal transition phenotype. This study investigated the role of PGCC formation in the prognostic value of neoadjuvant chemoradiation therapy (nCRT) in locally advanced rectal cancer (LARC). The morphological characteristics were observed in patients with LARC after nCRT. Colorectal cancer cell lines were treated with irradiation or chemotherapeutic drugs, and the metastasis-related proteins were detected. 304 nCRT cases and 301 paired non-nCRT cases were collected for analysis. More PGCCs and morphologic characteristics related to invasion and metastasis appeared in tumor tissue after nCRT. Irradiation or chemicals could induce the formation of PGCCs with daughter cells exhibiting strong migratory, invasive, and proliferation abilities. In patients after nCRT, pathologic complete remission, partial remission, stable disease, and progressive disease were observed in 29 (9.54%), 125 (41.12%), 138 (45.39%), and 12 (3.95%) patients, respectively. Mucinous adenocarcinomas (MCs) occurred more frequently in nCRT than in non-nCRT patients (*χ*^2^ = 29.352, *P*=0.001), and the prognosis in MC patients was worse than that in non-MC patients (*χ*^2^ = 24.617, *P*=0.001). The difference in survival time had statistical significance for 60 days (*χ*^2^ = 5.357, *P*=0.021) and 70 days (*χ*^2^ = 18.830, *P*=0.001) rest interval time. On multivariable analysis, 60 days rest interval, Duke's stage, and recurrence and/or distant metastasis remained significant predictors of survival. In conclusion, irradiation or chemicals induce the formation of PGCCs and PGCCs produce daughter cells with strong migration and invasion abilities after a long incubation period. Appropriate rest interval (incubation period) is very important for patients with LARC who will receive nCRT.

## 1. Introduction

In China, of those cancers that affect both men and women, colorectal cancer (CRC) is the second most common (12.2%) and rectal cancer ranks the seventh most common cause of cancer death [1]. Rectal cancer differs substantially from colon cancer, particularly in terms of clinical management [2]. Population-based cancer statistics provide an indicator of the overall effectiveness of the healthcare system in the context of effectiveness of screening, early diagnosis, and treatment services for patients with rectal cancer.

Polyploid giant cancer cells (PGCCs) are a special subpopulation of cancer cells that contribute to solid tumor heterogeneity. PGCCs differ from diploid cancer cells in size, tumorigenic ability, radioresistance, and chemoresistance. PGCCs possess functions of cancer stem cells and promote tumor maintenance and recurrence [3]. Our previous study showed that PGCC formation can be induced by CoCl_2_, paclitaxel, and other drugs. The polyploid nature of PGCCs was confirmed by fluorescence in situ hybridization and flow cytometry. Based on the long-term experimental data and observation, we defined the PGCC as a cancer cell that was at least three times larger in size than that of regular cancer cells [4–8]. PGCCs can generate daughter cells via asymmetric division, and these daughter cells show stronger migratory and invasive capacities than diploid cells, express less epithelial markers, and acquire mesenchymal phenotype [5, 7]. During the process of cancer development and progression, cancer cells gradually lose epithelial characteristics and acquire mesenchymal phenotype, called epithelial-mesenchymal transition, which is vital to cancer invasion and metastasis [9]. The number of PGCCs positively correlates with the malignant degree of cancer. In cancer, antimitotic chemotherapy drugs, radiotherapy, and hypoxia can increase the number of PGCCs. The formation of PGCCs can be involved in reactivation of embryonic developmental stages escaped from initial courses of treatment [10, 11]. Also, some other researchers have observed the rapid emergence of numerous PGCCs in a high-drug environment attributed to diploid epithelial cells converting to PGCCs, which suggests that PGCCs may be mediators of resistance in response to chemotherapeutic stress [12]. The number of PGCCs correlates with the recurrence, lymph node metastasis, chemoresistance, and poor prognosis of CRCs and is a good indicator to predict the metastasis and aggressiveness in CRCs [8].

Annually, more than 2000 radical CRC surgeries are performed at our hospital, of which 80% are performed for rectal cancer. For locally advanced rectal cancer (LARC), treatment includes neoadjuvant chemoradiation therapy (nCRT) followed by total mesorectal excision (TME) which comprises resection of the rectal tumor together with the fatty tissue surrounding the rectum [13], and this treatment methodology has been recommended for inclusion in clinical practice guidelines [14]. As of Aug 31, 2018, more than 300 LARC patients have received nCRT at our hospital. nCRT is currently the standard-of-care in stage II-III rectal cancer, resulting in tumor downstaging for patients with treatment-responsive disease. However, the prognosis of the downstaged patients treated with nCRT remains controversial. Furthermore, the optimal timing of surgery after nCRT (rest interval time) is unclear [13, 15, 16]. Rombouts et al. report that rest intervals of 9–12 weeks between surgery and CRT may improve the chances of pathologic complete response (pCR) in LARC patients, without an effect on overall survival (OS) [13]. Sun et al. reported that a rest interval of eight weeks after completion of nCRT appears to be the critical threshold for optimal tumor response [15]. Furthermore, prognoses in patients treated with nCRT and non-nCRT regimens have not been compared with a follow-up time of more than five years. The results of our study showed that differences in survival rates between patients treated with and without nCRT (nCRT and non-nCRT patients) increased gradually with time, when compared at three, five, six, seven, and eight years. A pathologic diagnosis of MC was more incident in nCRT cases, which may be related to the poor prognosis associated with nCRT.

In this study, we confirmed that irradiation and chemicals could induce the formation of PGCCs, and these PGCCs can generate daughter cells with strong migratory and invasive capacities. Furthermore, this paper also provides a comprehensive overview of nCRT in LARC, including the rest interval time, treatment response, and overall survival beyond five years, and discusses possible molecular mechanisms. A rest interval of less than 50 days may improve survival since nCRT may induce the formation of PGCCs and daughter cells with a strong migration and invasion capability; this may be related to the poor prognosis observed in patients treated with nCRT.

## 2. Materials and Methods

### 2.1. Culture of Cancer Cell Lines and Treatment

The human colorectal cancer cell lines LoVo and HCT116 were purchased from American Type Culture Collection (USA) and cultured in the RPMI-1640 medium supplemented with 10% fetal bovine serum, 100 U/mL penicillin, and 100 g/mL streptomycin. When the confluence of LoVo and HCT116 cells reached 90%, they were treated with radiation, capecitabine, oxaliplatin, and irinotecan. For irradiation, 9 Gy at a dose of 1.0 Gy/min with a ^137^Cs source was used. Detailed information about the chemotherapeutic drugs used is listed in Supplementary [Supplementary-material supplementary-material-1].

### 2.2. Hematoxylin-Eosin Staining and Immunocytochemical (ICC) Staining

The detailed information is provided in the supplementary Materials and Methods. In order to quantify protein expression of ICC, the sum of the staining intensity and the percentage of positive cell scores was performed to indicate the protein expression indexes for each section. The staining intensity was scored as follows: 0, no staining; 1, faint yellow staining; 2, moderate positive staining; and 3, strong positive staining. The percentage of positive cells was scored as follows: 0, <5% positive cells; 1, 6%–50% positive cells; 2, 51%–100% positive cells.

### 2.3. Plate Colony Formation Assay

The detailed information is provided in the supplementary Materials and Methods.

### 2.4. Wound-Scratch Assay, Cell Migration, and Invasion Assay

The detailed information is provided in the supplementary Materials and Methods.

### 2.5. Western Blotting

Western blot analyses were performed as described previously [5, 7]. Simply, total protein was extracted from the LoVo and HCT116 cells before and after treatment. The total protein was then separated on sodium dodecyl sulfate polyacrylamide gels and transferred to polyvinylidene fluoride membranes (Amersham Hybond-P PVDF Membrane; GE Healthcare). Information about the primary antibodies used is listed in Supplementary [Supplementary-material supplementary-material-1].

### 2.6. PGCC Definition and Counting

PGCCs are defined as a subpopulation of cancer cells with a nucleus that is at least three times larger than that of a regular diploid cancer cell, which was first described by Zhang et al. [7]. We counted the number of PGCCs per 100 tumor cells in five hot spots of each tumor sample. The size of each PGCC nucleus was measured using a micrometer. The average PGCCs number per 100 tumor cells was calculated for statistical analyses.

### 2.7. Patient Population

LARC patients (*n* = 605) (T3/4 and/or N1 disease confirmed by magnetic resonance imaging, MRI), including patients treated with neoadjuvant chemoradiation (nCRT) (*n* = 304) and those not treated with nCRT (non-nCRT patients) (*n* = 301), followed by surgery (radical or palliative operation) and postoperative chemotherapy at the Tianjin Union Medical Center between 2009 and 2018, were enrolled. The paired 301 non-nCRT patients who did not receive nCRT, mainly due to the poor compliance, such as financial burden, traffic inconvenience for outside patients, and nCRT-refusal patients. All patients had complete pathologic or survival data. The follow-ups of all these patients were completed in August 2018. Data of patients with surgery completed prior to August 30, 2015, were used for survival analysis. Furthermore, paraffin-embedded tissue samples with locally advanced rectal cancer after nCRT were obtained from the Tumor Tissue Bank of Tianjin Union Medical Center. Morphologic characteristics were observed in the liver metastases, and recurrence was seen in some patients. Furthermore, the 301 non-nCRT patients were used as paired controls and were matched based on the MRI stage, tumor differentiation, sex, operation time, surgical procedures, and age (the difference in age of one-by-one matched patient between the nCRT and non-nCRT was less than 5 years), to minimize potential for bias. Three patients in the nCRT group were not paired because of lack of patients who met the inclusion criteria. There was no history of inflammatory bowel disease and familial colorectal cancer in these patients. Patients with perioperative mortalities were also excluded. The clinicopathologic data for each patient, including age, sex, MRI stage, start and end time of nCRT, tumor characteristics, surgery details, administration of postoperative chemotherapy, date of the last follow-up, date of recurrence, and date of death, were collected. This study was approved by the Hospital Review Board, and the confidentiality of patient information was maintained.

### 2.8. Treatment Characteristics

All patients in the nCRT and non-nCRT groups received surgery and/or nCRT at our hospital. At our hospital, nCRT is offered to most patients with MRI stage II (T3-4, node-negative disease with tumor penetration through the muscle wall) or MRI stage III (node positive disease without distant metastasis) disease according to the National Comprehensive Cancer Network (NCCN) Clinical Practice Guidelines in Oncology [14, 17]. All the 605 patients were offered adjuvant chemotherapy after surgery.

### 2.9. Neoadjuvant Chemoradiation

The nCRT regimen for rectal cancer consists of chemotherapy combined with medium-dose radiotherapy before surgery. Radiotherapy for rectal cancer was performed with three-dimensional conformal radiotherapy (3DCRT) or intensity-modulated radiotherapy (IMRT). After affixing a body mask, computed tomography (CT) scans were performed on patients in a prone or supine position. The CT images were then transmitted to the radiotherapy planning system for three-dimensional reconstruction and target mapping. Gross tumor volume (GTV) included primary tumors and metastatic lymph nodes. Clinical target volumes (CTV) included the perimesenteric lymphatic drainage area, obturator lymphatic drainage area, and the iliac lymphatic drainage area. The presacral lymphatic drainage area, if necessary, included the external iliac lymph drainage area. Radiation doses were as follows: planning target volume (PTV), 45 Gy in 25 fractions to the pelvis, and gross tumor volume (GTV), 48 Gy in 25 fractions to the tumor. Patients were irradiated for five weeks, five times a week, on weekdays. Chemotherapy was administered for five weeks and synchronized with radiotherapy. The patients were treated with capecitabine monotherapy or with two cycles of the CAPOX regimen (capecitabine (trade name Xeloda) combined with oxaliplatin). All patients who received adjuvant chemotherapy were examined by a medical oncologist. The final decision and choice of regimen was made on an individualized basis.

### 2.10. Scoring Pathologic Response

Pathologic responses to nCRT were scored using the American Joint Committee on Cancer (AJCC) and the College of American Pathologists (CAP) guidelines [18], defined as follows: complete response (CR), no viable cancer cells; partial response (PR), single or small groups of cancer cells; minimal response (stable disease, SD), residual cancer outgrown by fibrosis; poor response (progressive disease, PD), minimal or no tumor kill, and extensive residual cancer [19, 20]. Scoring was performed by two pathologists at our hospital who were blinded to the clinical treatments.

### 2.11. Statistical Analyses

Statistical software SPSS 17.0 (IBM Corporation, USA) was used to evaluate the data, and a 2-tailed *P* value of less than 0.05 was defined as statistically significant. Pearson's chi-square (*χ*^2^) test was used to analyze the differences in clinicopathologic characteristics between patients in the nCRT and non-nCRT groups. Survival time was analyzed using the Kaplan–Meier method, and differences were assessed using the log-rank test.

## 3. Results

### 3.1. Morphologic Observation in CRCs after nCRT

**Figure 1:**
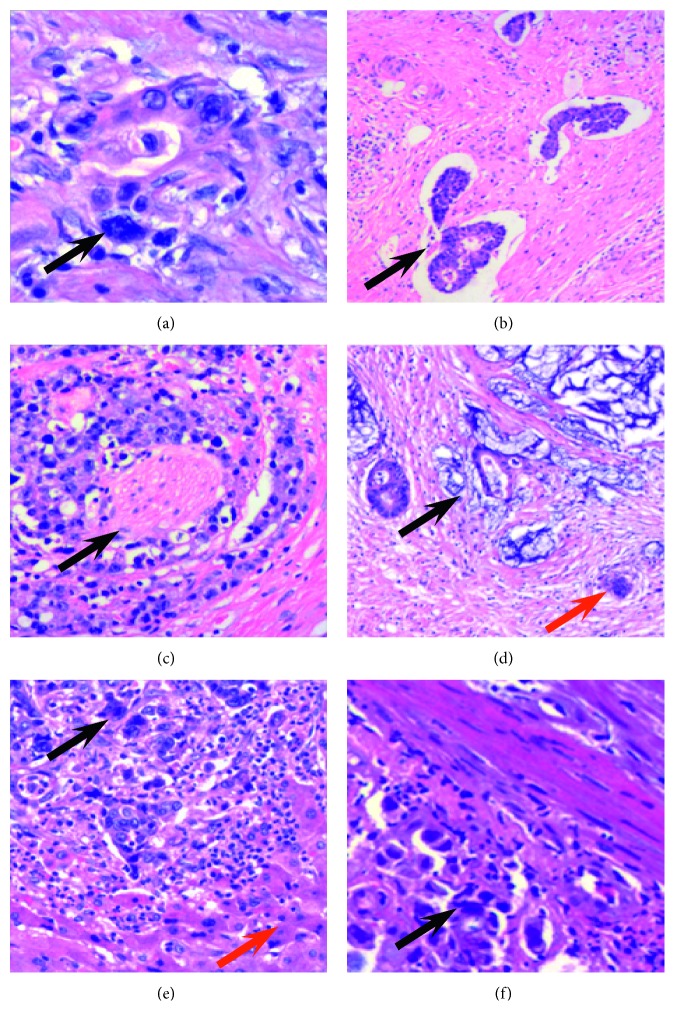
The number of PGCCs increased in colorectal cancers (CRCs) after neoadjuvant chemoradiation therapy (nCRT). (a) Many PGCCs appeared in the tumor after nCRT (black arrow heads, H&E, 100x). (b) Tumor emboli (black arrow heads, H&E, 100x). (c) Perineural invasion of cancer cells (black arrow heads, H&E, 100x). (d) Mucinous adenocarcinomas (black arrow heads) and PGCCs (red arrow heads) occurred after nCRT (H&E, 100x). (e) Many PGCCs (black arrow heads) appeared in the liver (red arrow heads represent the liver tissue) metastatic rectal cancer in patients after nCRT (H&E, 100x). (f) Many PGCCs appeared in the anastomotic recurrence in patients with locally advanced rectal cancer after nCRT (black arrow heads, H&E, 100x).

Many PGCCs appeared in tumor tissue after nCRT (Figure 1(a)), and different morphologic characteristics related with tumor invasion and metastasis in cancer cells appeared in tumor tissue after nCRT. The average number of PGCCs per 100 tumor cells for all tumor tissues in nCRT (exclusion of pathologic CR patients in nCRT) was significantly more than that in non-CRT patients (*F* = 35.38, *P*=0.001; Table 1). Tumor emboli in blood or lymphatic vessels (Figure 1(b)) and perineural invasion of cancer cells (Figure 1(c)) could also be observed in tumor tissue after nCRT. The incidence of tumor emboli was higher in nCRT patients than in non-nCRT patients (*χ*^2^ = 5.117, *P*=0.025; Table 2). Furthermore, as described above, MC occurred more frequently in nCRT than in non-nCRT patients (*χ*^2^ = 29.352, *P*=0.001; Table 2). Mucus often appeared in the cytoplasm of cancer cells after nCRT (Figure 1(d)). There were more PGCCs appearing in the liver metastatic rectal cancer than the primary tumor (Figure 1(e)). More PGCCs were observed in anastomotic recurrent rectal cancer after nCRT than before nCRT (Figure 1(f)). In the tumor tissues, PGCCs were usually located in the necrosis margin and infiltrating front, resulting from the environment of PGCCs formation.

### 3.2. Formation of PGCCs in Response to Radiation and Chemotherapeutics Treatment In Vitro

**Figure 2:**
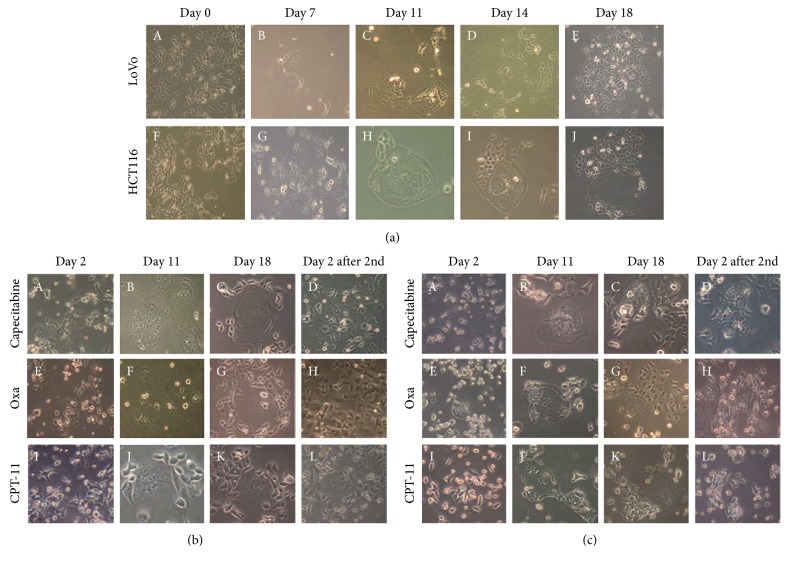
(a) Time-lapse observation of cultured LoVo and HCT116 cells after irradiation (40x): (A and F) morphologic characteristics of LoVo and HCT116 before irradiation (B and G), 7 days after irradiation (C and H), 11 days after irradiation (D and I), 14 days after irradiation (E and J), and 18 days after irradiation. (b) Time-lapse observation of LoVo after chemotherapeutic treatment (40x): (A) 2 days after capecitabine treatment, (B) 11 days after capecitabine treatment, (C) 18 days after capecitabine treatment, (D) 2 days after a second capecitabine treatment, (E) 2 days after oxaliplatin treatment, (F) 11 days after oxaliplatin treatment, (G) 18 days after oxaliplatin treatment, (H) 2 days after a second oxaliplatin treatment, (I) 2 days after irinotecan treatment, (J) 11 days after irinotecan treatment, (K) 18 days after irinotecan treatment, and (L) 2 days after a second irinotecan treatment. (c) Time-lapse observation of HCT116 after chemotherapeutic treatment (40x): (A) 2 days after capecitabine treatment, (B) 11 days after capecitabine treatment, (C) 18 days after capecitabine treatment, (D) 2 days after a second capecitabine treatment, (E) 2 days after oxaliplatin treatment, (F) 11 days after oxaliplatin treatment, (G) 18 days after oxaliplatin treatment, (H) 2 days after a second oxaliplatin treatment, (I) 2 days after irinotecan treatment. (J) 11 days after irinotecan treatment, (K) 18 days after irinotecan treatment, and (L) 2 days after a second irinotecan treatment.

Colon cancer cell lines LoVo and HCT116 were cultured in medium and treated with radiation and chemotherapeutics (capecitabine, oxaliplatin, and irinotecan) when the confluency reached 90%. Two days after treatment with radiation (Figure 2(a)-A and F) and chemotherapeutics (Figures 2(b)-A, E, and I and 2(c)-A, E, and I), most diploid LoVo and HCT116 cells died, whereas scattered PGCCs could be clearly visualized after removing floating dead cells. A PGCC was defined as a tumor cell with a nucleus at least 3 times larger than that of a diploid tumor cell, and such cells exhibited properties of cancer stem cells [7]. As described in our previously published papers [5, 7, 8], LoVo and HCT116 PGCCs treated with radiation and chemotherapeutic agents can generate small daughter cells through asymmetric cell division. The cellular dynamics of the radiation-treated LoVo and HCT116 were recorded with a microscope over the course of 18 days in a fixed field (Figures 2(a)–2(c)). Eighteen days (incubation period) after radiation, single PGCCs generated hundreds of daughter cells (Figures 2(a)-E and J; 2(b)-C, G, and K; 2(c)-C, G, and K). These cells recovered from the treatment with radiation and chemotherapeutics once and were then treated for the second time. Two days after the second treatment, only a few cells died, while most cells survived the treatment (Figures 2(b)-D, H, and I and 2(c)-D, H, and L); this meant that cells which recovered from the first treatment were resistant to the second treatment.

### 3.3. PGCCs and Their Daughter Cells Exhibit Strong Migration, Invasion, and Proliferation Capabilities

**Figure 3:**
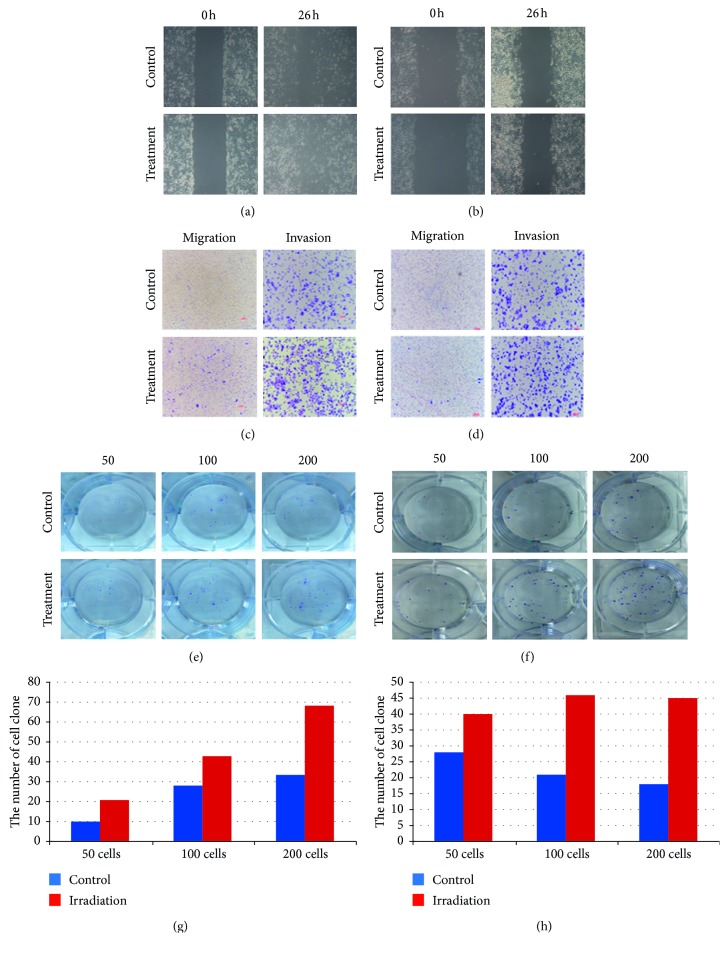
(a) Wound-scratch assay of LoVo cells at 0 hours and 26 hours after irradiation (40x). (b) Wound-scratch assay of HCT116 cells at 0 hours and 26 hours after irradiation (40x). (c) Migration and invasion assay of LoVo cells before and after irradiation (40x). (d) Migration and invasion assay of HCT116 cells before and after irradiation (40x). (e) Plate colony formation assay of 50, 100, and 200 LoVo cells/well before and after irradiation (40x). (f) Plate colony formation assay of 50, 100, and 200 HCT116 cells/well before and after irradiation (40x). (g) Bar graph depicting the number of clones in wells containing 50, 100, and 200 LoVo cells/well, before and after irradiation. (h) Bar graph depicting the number of clones in wells containing 50, 100, and 200 LoVo cells/well, before and after irradiation.

To determine whether PGCCs and their daughter cells had stronger migration, invasion, and proliferation capabilities than did the control cells, wound healing, cell migration, and invasion assays using matrigel-coated transwell inserts and plate colony formation assays were performed. Figures 3(a) and 3(b) depict the results of the wound-scratch assay at various incubation periods. The spaces covering the scratched surface in the panels were observed to gradually become narrower at 26 h incubation periods. The cell migration and invasion capabilities of PGCCs and their daughter cells were found to increase. Moreover, results of the transwell migration and invasion assays showed that compared to control cells, a higher number of treated PGCCs and their daughter cells showed migration and invasion capabilities (Figures 3(c) and 3(d)). Furthermore, we examined the proliferation abilities of cells before and after treatment. Results of the plate colony formation assay showed that cells subjected to treatment exhibited more clone formation compared to control cells (Figures 3(e) and 3(f)). Figures 3(g) and 3(h) show that the number of clones increased with increasing incubation time.

### 3.4. PGCCs and Their Daughter Cells Exhibit a Mesenchymal Phenotype

**Figure 4:**
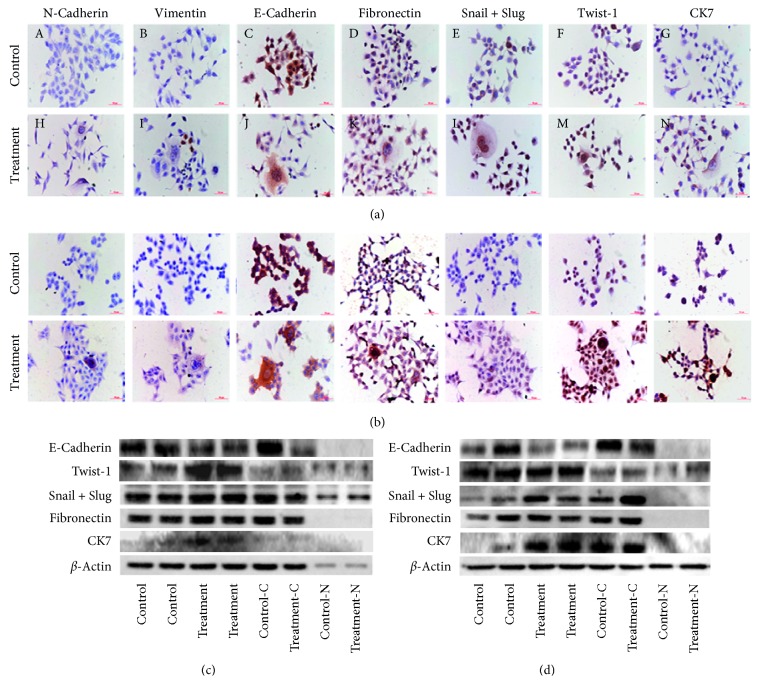
(a) Immunocytochemical (ICC) staining of N-cadherin, vimentin, E-cadherin, Fibronectin, Snail and Slug, Twist-1, and CK7 in LoVo cells before and after irradiation (100x). (b) ICC staining of N-cadherin, vimentin, E-cadherin, fibronectin, Snail and Slug, Twist-1, and CK7 in HCT116 cells before and after irradiation (100x). (c) Western blot assay of E-cadherin, Twist-1, Snail and Slug, fibronectin, and CK7 expression in LoVo cells before and after irradiation (-C, -cytoplasm; -N, -nuclear; 100x). (d) Western blot assay of E-cadherin, Twist-1, Snail and Slug, fibronectin, and CK7 expression in HCT116 cells before and after irradiation (-C, -cytoplasm; -N, -nuclear; 100x).

PGCCs with budding daughter cells and the control cells were cultured on coverslips for ICC staining. Results of E-cadherin, N-cadherin, vimentin, fibronectin, Snail and Slug, Twist-1, and CK7 staining showed that the control LoVo cells were negative for N-cadherin and vimentin. A few PGCCs and their daughter cells were positive for N-cadherin (Figures 4(a)-A and 4(b)-A) and vimentin (Figures 4(a)-B, 4(b)-B). E-cadherin expression in the control cells was higher than that in treated cells (Figures 4(a)-C and 4(b)-C). The expression levels of fibronectin, Snail and Slug, Twist-1, and CK7 in the LoVo and HCT116 PGCCs and their daughter cells were higher than those in the control cells (Figures 4(a) and 4(b)). The quantitative results of N-cadherin, Vimentin, E-cadherin, Fibronectin, Snail + Slug, Twist-1, and CK7 of ICC staining in PGCCs with budding daughter cells and the control cells of LoVo and HCT116 are showed in Supplementary Figures [Supplementary-material supplementary-material-1] and [Supplementary-material supplementary-material-1]. Furthermore, the subcellular location of Slug and Snail and Twist after treatment was different from that in the control cells. The nuclei of PGCCs were positive for Slug and Snail and Twist with ICC staining. Western blot analysis confirmed greater levels of Slug and Snail, Twist, and CK7 expression in PGCCs and in their budding daughter cells than those in control cells. The expression level of E-cadherin in control cells was higher than that in the PGCCs and their budding daughter cells (Figures 4(c) and 4(d)).

### 3.5. Comparison of Long-Term Follow-Up Prognosis in Patients with Locally Advanced Rectal Cancer with and without nCRT

Six hundred and five LARC cases (T3/4 and/or N1) including 304 patients treated with nCRT and 301 patients treated without nCRT followed by surgery were analyzed. The mean patient age at the time of surgery was 60.21 years (range 29–84 years), and 32.23% were women. For patients treated with nCRT, the mean patient age at surgery was 60.02 years (range 32–84 years) and 32.24% (98/304) were women. Radical surgery was performed in 292 patients, and palliative operation was performed in 12 patients. For the paired group (non-nCRT group), the mean age at surgery was 60.40 years (range 29–83 years), and 32.22% (97/304) were women. After nCRT, of 304 patients, 29 patients (9.54%) achieved pathologic CR, and 125 (41.12%), 138 (45.39%), and 12 (3.95%) patients achieved PR, SD, and PD, respectively. R0 resection was not performed in 12 patients (3.95%). Clinicopathologic characteristics of patients treated with nCRT are summarized in Table 3. After surgery (radical and palliative surgeries), all 605 patients received adjuvant chemotherapy. In the nCRT group, therapeutic response did not differ significantly based on age, sex, and histological differentiation. The differences in therapeutic response showed statistically significant differences as per T stage (*χ*^2^ = 440.12, *P*=0.001) and N stage (*χ*^2^ = 18.007, *P*=0.001). After nCRT, the prognosis of MC cases was worse than that of non-MC cases (*χ*^2^ = 24.617, *P*=0.001).

**Figure 5:**
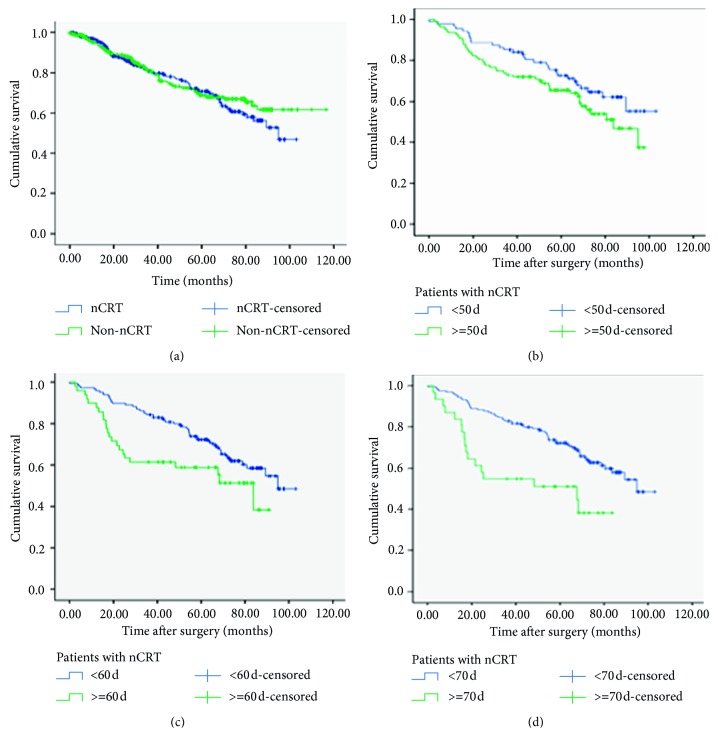
(a) Comparison of overall survival in patients treated with neoadjuvant chemoradiation therapy (nCRT) and those not treated with nCRT (non-nCRT). (b) Comparison of overall survival in nCRT patients with less than 50 days rest interval and no less than 50 days rest interval. (c) Comparison of overall survival in nCRT patients with less than 60 days rest interval and no less than 60 days rest interval. (d) Comparison of overall survival in nCRT patients with less than 70 days rest interval and no less than 70 days rest interval.

**Figure 6:**
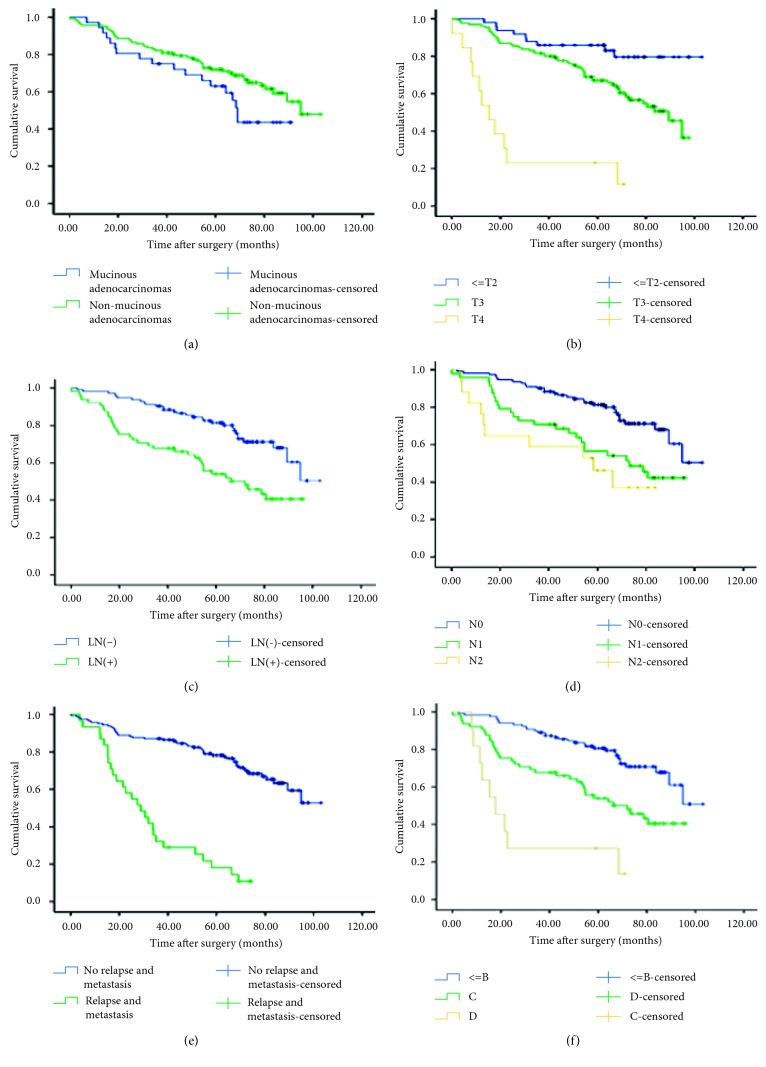
(a) Overall survival of patients with mucinous adenocarcinoma and nonmucinous adenocarcinoma after neoadjuvant chemoradiation therapy (nCRT). (b) Overall survival of in patients at different T stages (T1 + T2 vs. T3 + T4) after nCRT. (c) Overall survival of patients with and without lymph node metastasis after nCRT. (d) Overall survival of patients with N0, N1, and N2 disease after nCRT. (e) Overall survival of nCRT patients with positive recurrence and/or distant metastasis and nonpositive recurrence and/or distant metastasis. (f) Overall survival of nCRT patients at different Duke's stages.

There was no significant difference in the survival rate between the nCRT and non-nCRT groups. However, differences in survival rates increased gradually over time between the nCRT and non-nCRT groups (survival rates: three year (*χ*^2^ = 0.075, *P*=0.784), five year (*χ*^2^ = 0.094, *P*=0.759), six year (*χ*^2^ = 0.722, *P*=0.396), seven year (*χ*^2^ = 1.376, *P*=0.241), and eight year (*χ*^2^ = 2.995, *P*=0.084) (Table 4) (Figure 5(a)). Furthermore, survival time in the nCRT group was associated with the rest interval. With a rest interval time of less than 50 days, the difference in survival time was not statistically significant (*χ*^2^ = 2.634, *P*=0.105) (Figure 5(b)). The difference in survival time was statistically significant for a rest interval time of 60 days (*χ*^2^ = 5.357, *P*=0.021, Figure 5(c)) and 70 days (*χ*^2^ = 10.830, *P*=0.001, Figure 5(d)) (Table 5). MC after surgery was more frequent in the nCRT group than it was in the non-nCRT group, and the difference was statistically significant (*χ*^2^ = 29.352, *P*=0.001) (Table 2) (Figure 6(a)). There were no differences in lymph node metastasis rates between the nCRT and non-nCRT groups (Table 2).

The association of clinicopathologic and treatment characteristics with overall survival (OS) is detailed in Table 6. Response to nCRT in the nCRT group was associated with OS on univariable analysis (log rank). pCR, pPR, and pSD were significantly different between the study groups, and hence, these parameters were grouped together and compared against pPD during analysis (*χ*^2^ = 7.773, *P*=0.005). More than 60 days rest interval (*χ*^2^ = 5.357, *P*=0.021), higher T stage (Tis + T1 + T2 vs. T3: *χ*^2^ = 7.553, *P*=0.006, T3 vs. T4: *χ*^2^ = 30.120, *P*=0.001, Tis + T1 + T2 vs. T4: *χ*^2^ = 39.765, *P*=0.001) (Figure 6(b)), positive lymph node metastasis (*χ*^2^ = 13.722, *P*=0.001) (Figures 6(c) and 6(d)), positive recurrence and/or distant metastasis (*χ*^2^ = 66.532, *P*=0.001) (Figure 6(e)), and higher Duke's stage (A + B vs. C: *χ*^2^ = 13.527, *P*=0.001, C vs. D: *χ*^2^ = 7.415, *P*=0.006, A + B vs. D: *χ*^2^ = 38.719, *P*=0.001) (Figure 6(f)) were associated with poor survival. On multivariable (Cox proportional hazards) analysis, rest interval (60 days), Duke's stage, and recurrence and/or distant metastasis remained significant predictors of survival (Table 6).

## 4. Discussion

Tumor invasion and metastasis are the main causes of tumor recurrence and patient mortality [21]. Treatments used for CRC include surgery, chemotherapy, radiation, and targeted therapy. For patients with LARC, nCRT has become the standard treatment strategy over the past decade [22] and has been recommended by major guidelines [14]. Our previous studies have confirmed that chemotherapeutic drugs could induce the formation of PGCCs, and PGCCs can produce daughter cells with strong abilities of tumor infiltration and metastasis in vitro [4, 5, 7, 23, 24]. Morphologic observation of human locally advanced rectal cancer after nCRT showed that there were many PGCCs in tumor tissue after nCRT and different morphologic characteristics related with tumor invasion and metastasis appeared in tumor tissue. In this study, we showed that irradiation and chemicals could induce the formation of PGCCs in vitro and PGCCs with their generated daughter cells exhibited strong migratory, invasive, and proliferation abilities, associating with the therapeutic effect of nCRT for LARC patients.

Most clinical studies have shown that the main benefits of nCRT lie in improving staging and cancer resectability though long-term OS and progression-free survival are not affected significantly [25]. At present, the most commonly used nCRT is radiotherapy and synchronized adjuvant chemotherapy for five weeks followed by surgery 6–8 weeks after radiotherapy [26]. However, different rest interval time between radiotherapy and surgery may affect the survival of patients with nCRT [3]. Several clinical studies have suggested that prolonging the time interval may help to increase the rate of pathologic CR and reduce the clinical stage. Retrospective studies have reported that a 9–12-week rest interval time after nCRT can improve the rate of pCR in LARC patients though there is no effect on OS. Compared with <8 weeks rest interval time, >8 weeks rest interval time after CRT can reduce tumor staging and increase pCR rate [27]. In 2016, the European Society for Medical Oncology (ESMO) reported a randomized controlled study of more than 200 patients. Compared with those who had a 6-week rest interval time, the pCR rate in patients with a 12-week rest interval time was significantly higher and the mrT staging was significantly lower [28]. However, these studies have focused on pathological remission or a decline in tumor staging, even though pCR or magnetic resonance tumor regression grade (mrTRG) is only a predictor of long-term prognosis. Differences in OS and disease-free survival have nevertheless been observed among patients after nCRT [29, 30]. Patients with a long rest interval time after nCRT therapy may have a higher rate of pCR, but the survival benefits need to be studied with a long-term follow-up. Another randomized controlled study comparing 11 weeks and 7 weeks of rest interval time showed that there was no statistically significant difference in pCR rates. In contrast, patients with 11 weeks of rest interval time had a higher incidence of complications and incomplete mesorectal excisions, which may be related to difficulties during surgery after a long rest interval time [31]. Another retrospective study of more than 10,000 nCRT-treated cases showed that 8 weeks of rest interval time may be optimum and the risk of positive margins increased when the rest interval time was more than 8 weeks [15]. Kwak et al. studied 1785 cases of LARC and found that the rate of tumor downstaging peaked at 6-7 weeks of rest interval after nCRT and decreased thereafter [32]. Here, by analyzing the clinicopathologic characteristics of 304 patients treated with nCRT and those of paired 301 patients not treated with nCRT, we concluded that the survival time of LARC patients treated with nCRT was related to the rest interval. For a rest interval time of more than 50 days, the difference in survival time was not statistically significant. When the rest interval time was more than 60 days, the difference in survival time was statistically significant. Results of multivariable analysis showed that 60 days rest interval, Duke's stage, and recurrence and/or distant metastasis remained significant predictors of survival. Furthermore, in patients after nCRT, MC (a highly metastatic malignant tumor) occurred more frequently in nCRT after surgery than in non-nCRT patients. MC has been reported to be associated with a higher risk of death when located in the rectum [33]. The differences in survival rates between the nCRT and non-nCRT groups increased gradually with time, though the differences in three-year, five-year, six-year, seven-year, and eight-year survival rates were not significant.

To further study the possible mechanisms by which nCRT affects the survival of patients with LARC, chemotherapeutic drugs and irradiation were used to treat colon cancer cell lines LoVo and HCT116. Results showed that chemotherapeutic drugs and irradiation can induce the formation of PGCCs. We have previously demonstrated that PGCCs induced with CoCl_2_ and paclitaxel exhibit cancer stem cell properties and asymmetrically generate daughter cells via budding [5–7]. The number of PGCCs correlates positively with the degree of malignancy. Presence of PGCC-enriched tumor tissue correlates with a high recurrence rate, lymph node metastasis, chemoresistance, and poor prognosis. Furthermore, daughter cells produced by PGCCs via asymmetric cell division have strong migration and invasion capabilities [5–7, 34]. In contrast to control cells, daughter cells generated by PGCCs after irradiation and chemicals acquired a mesenchymal phenotype and expressed epithelial-mesenchymal transition-related proteins including N-cadherin, vimentin, Twist, Slug, Snail, and CK7.

PGCCs have been reported in different types of cancer cell lines including those of ovarian cancer [7], colon cancer [35], and breast cancer [36]. After chemotherapeutic drug and radiation treatment, a long incubation period is required before daughter cells are generated and the length of this period depends on the chemotherapy dose and intensity of radiotherapy. We previously treated SKOv3 cells with1 *μ*M paclitaxel for 48 hours and observed that SKOv3 PGCCs generated daughter cells four months later. The long incubation period may be related to the increasing differences in survival rates of the nCRT and non-nCRT groups observed in this study at three-year, five-year, six-year, seven-year, and eight-year time periods. Furthermore, when cells were treated with chemotherapeutic drugs and radiation for the first time, the surviving PGCCs recovered and generated daughter cells, which were resistant to a second treatment with the same dose of chemotherapeutic drugs and intensity of irradiation. Compared with first time treatment, more PGCCs survived and the recovery time was also shortened after the second treatment.

## 5. Conclusions

Taken together, compared to patients not treated with nCRT, those treated with nCRT did not show improved long-term survival rates in LARC. The detailed molecular mechanisms by which chemotherapeutic drugs and irradiation induce the formation of PGCCs with daughter cells resistant to treatment need to be studied further.

## Figures and Tables

**Table 1:** Comparison of the average number of PGCCs per 100 tumor cells in patients treated with neoadjuvant chemoradiation therapy (nCRT) and without nCRT.

Group	*n*	Average number of PGCCs per 100 tumor cells	Value of statistic	*P*
nCRT	275	11.5 ± 6.34	*F* = 35.38	0.001
Non-nCRT	301	3.7 ± 1.54		

nCRT, neoadjuvant chemoradiation therapy.

**Table 2:** Comparison of the incidence of tumor emboli, histological type, and lymph node metastasis in patients treated with neoadjuvant chemoradiation therapy (nCRT) and without nCRT.

Group	nCRT	Non-nCRT	Chi-square	*P*
Tumor emboli	198	169	5.117	0.025
Nontumor emboli	106	132		
Mucinous adenocarcinoma	59	16	29.352	0.001
Nonmucinous adenocarcinoma	219	267		
Lymph node metastasis	184	163	0.929	0.377
Nonlymph node metastasis	99	104		

nCRT, neoadjuvant chemoradiation therapy.

**Table 3:** Clinicopathological characteristics in 304 cases of locally advanced rectal cancer patients treated with neoadjuvant chemoradiation therapy (nCRT).

	AJCC and CAP regression score					Chi-square	*P* value
	Total	CR	PR	SD	PD		
Number of patients	304	29 (9.54%)	125 (41.12%)	138 (45.39%)	12 (3.95%)		
Age (years), mean	60.02	61.09	59.40	59.61	66.58	4.830	0.185
Sex							
Male	206 (67.76%)	20 (9.71%)	85 (41.26%)	92 (44.66%)	9 (4.37%)		
Female	98 (32.24%)	9 (9.18%)	40 (40.82%)	46 (46.94%)	3 (3.06%)	0.3355	0.953
Rest interval (d), mean	68.46	69.10	55.29	72.97	152.17	6.733	0.081
Pathology N stage^◆^						440.120	0.001
Complete response	27 (8.91%)	25 (92.59%)	2 (7.41%)	0	0		
T1	9 (2.97%)	0	8 (88.89%)	1 (11.11%)	0		
T2	43 (14.19%)	1 (2.33%)	25 (58.14%)	17 (39.53%)	0		
T3	197 (65.02%)	3 (1.52%)	83 (42.13%)	111 (56.35%)	0		
T4	27 (8.91%)	0	6 (22.22%)	9 (33.33%)	12 (44.44%)		
Pathology N stage^*∗*^						18.007	0.001
N0	184 (65.02%)	27 (14.67%)	80 (43.48%)	77 (41.85%)	0		
N1	72 (25.44%)	0	25 (34.72%)	47 (65.28%)	0		
N2	27 (9.54%)	2 (7.41%)	12 (44.44%)	13 (48.15%)	0		
Histological type^▼^						24.617	0.001
Mucinous carcinoma	59 (21.22%)	11 (18.64%)	27 (45.76%)	21 (35.59%)	0		
Nonmucinous carcinoma	219 (78.78%)	5 (2.28%)	97 (44.29%)	117 (53.42%)	0		
Histological differentiation^△^						1.781	0.411
Well + moderately	190 (88.79%)	3 (1.58%)	86 (45.26%)	101 (53.16%)	0		
Poor	24 (11.21%)	0	8 (33.33%)	16 (66.67%)	0		

^◆^1 missing value; ^*∗*^21 missing values; ^▼^26 missing values; ^△^90 missing values. CR, complete remission; PR, partial remission; PD, progressive disease; SD, stable disease; AJCC, American Joint Committee on Cancer; CAP, College of American Pathologists; nCRT, neoadjuvant chemoradiation therapy.

**Table 4:** Comparison of survival rate of locally advanced rectal cancer in patients treated with neoadjuvant chemoradiation therapy (nCRT) and without nCRT.

Group	3-year	5-year	6-year	7-year	8-year
nCRT	78.35% (153/195)	64.02% (105/164)	48.91% (67/137)	29.81% (31/104)	6.25% (5/80)
Non-nCRT	79.64% (133/167)	62.32% (86/138)	54.24% (64/118)	37.78% (34/90)	17.54% (10/57)
Chi-square	0.075	0.094	0.722	1.376	2.995
*P* value	0.784	0.759	0.396	0.241	0.084

nCRT, neoadjuvant chemoradiation therapy.

**Table 5:** Comparison of survival in patients with different days to surgery after neoadjuvant chemoradiotherapy for locally advanced rectal cancer.

Group	*n*	Dead	Alive	Chi-square	*P*
<50 days	88	30	58	2.634	0.105
≥50 days	107	46	61		
<60 days	146	53	93	5.357	0.021
≥60 days	49	23	26		
<70 days	164	59	105	10.830	0.001
≥70 days	31	17	14		

**Table 6:** Kaplan–Meier analysis of locally advanced rectal cancer patients treated with neoadjuvant chemoradiation (nCRT).

Variable	Mean Survival (months)	Chi-square	*P*	Survival analysis (Cox)			
				B	OR	95% CI	*P*
Rest interval							
≥60 days	46.35 ± 27.99	5.357	0.021	0.612	1.845	1.031–3.302	0.039
<60 days	61.39 ± 24.13						
Pathological stage							
Tis + T1 + T2	64.42 ± 22.53^■^	7.553	0.006				
T3	58.69 ± 24.61^□^	30.120	0.001				
T4	24.57 ± 24.61^▲^	39.765	0.001				
Duke's stage							
A + B	62.97 ± 21.60^△^	13.527	0.001				
C	52.69 ± 29.32^▼^	7.415	0.006	0.409	1.505	1.174–1.930	0.001
D	28.65 ± 24.6^▽^	38.719	0.001				
nCRT							
CR + PR + SD		7.773	0.005				
PD							
LN metastasis							
No	63.45 ± 21.30	13.722	0.001				
Yes	52.69 ± 29.32						
Relapse and/or distant metastasis		66.532	0.001	1.839	6.293	3.591–11.027	0.001
No	62.21 ± 24.15						
Yes	33.28 ± 21.18						

^■^Compared with T3; ^□^compared with T4; ^▲^compared with Tis + T1 + T2; ^△^compared with C; ^▼^compared with D; ^▽^compared with A + B. OR, odds ratio; CI, confidence interval; nCRT, neoadjuvant chemoradiation therapy; CR, complete remission; PR, partial remission; PD, progressive disease; SD, stable disease; LN, lymph node; nCRT, neoadjuvant chemoradiation therapy.

## Data Availability

All data supporting the findings of this study are available within the article and its supplementary files and are available from the corresponding author upon reasonable request.

## Abbreviations

LARC: Locally advanced rectal cancer
CRT: Neoadjuvant chemoradiation therapy
MC: Mucinous adenocarcinomas
PGCCs: Polyploid giant cancer cells
CoCl_2_: Cobalt chloride
CRC: Colorectal cancer
TME: Total mesorectal excision
pCR: Pathologic complete response
OS: Overall survival
PR: Partial response
SD: Stable disease
PD: Progressive disease
ICC: Immunocytochemical
WB: Western blot
GTV: Gross tumor volume
CTV: Clinical target volumes
PTV: Planning target volume
mrTRG: Magnetic resonance tumor regression grade.

## Supplementary Materials

Supplementary Materials Supplementary Figure S1: quantitative results of the expression differences of N-cadherin, vimentin, E-cadherin, fibronectin, Snail + Slug, Twist-1, and CK7 in LoVo control cells and treatment cells are shown as histograms. Each bar represents the mean ± SD of three independent experiments (^*∗*^*P* < 0.05). Supplementary Figure S2: quantitative results of the expression differences of N-cadherin, vimentin, E-cadherin, fibronectin, Snail + Slug, Twist-1, and CK7 in HCT116 control cells and treatment cells are shown as histograms. Each bar represents the mean ± SD of three independent experiments (^*∗*^*P* < 0.05). Supplementary Table S1: different concentrations and durations of chemotherapeutic treatment in different cell lines. Supplementary Table S2: detailed information of antibodies used in this study.
