# Supplementary material for: Formation of Polyploid Giant Cancer Cells Involves in the Prognostic Value of Neoadjuvant Chemoradiation in Locally Advanced Rectal Cancer
Source: J Oncol. 2019 Aug 29;2019:2316436. doi: 10.1155/2019/2316436 (PMC6735173; doi:10.1155/2019/2316436)
Supplement: Supplementary Materials — Supplementary Figure S1: quantitative results of the expression differences of N-cadherin, vimentin, E-cadherin, fibronectin, Snail + Slug, Twist-1, and CK7 in LoVo control cells and treatment cells are shown as histograms. Each bar represents the mean ± SD of three independent experiments (∗P < 0.05). Supplementary Figure S2: quantitative results of the expression differences of N-cadherin, vimentin, E-cadherin, fibronectin, Snail + Slug, Twist-1, and CK7 in HCT116 control cells and treatment cells are shown as histograms. Each bar represents the mean ± SD of three independent experiments (∗P < 0.05). Supplementary Table S1: different concentrations and durations of chemotherapeutic treatment in different cell lines. Supplementary Table S2: detailed information of antibodies used in this study. [file 2316436.f1.doc]

**Supplementary Materials and Methods**

**Hematoxylin-eosin staining and immunocytochemical (ICC) staining**

Sections of 4 µm thickness obtained from formalin-fixed, paraffin-embedded spheroid tissues were deparaffinized, rehydrated, and counterstained with hematoxylin for 3 min and with eosin for 2 min. The sections were then dehydrated and mounted on coverslips. For immunocytochemical (ICC) staining, cells were grown on glass coverslips before and after treatment until 90% confluence, and were then fixed with 75% ethanol. After washing with PBS, these slides were incubated overnight with different primary antibodies at 4°C (antibody information is listed in supplementary Table-2). Finally, the cells were counterstained with hematoxylin and examined under a microscope.

**Plate colony formation assay**

Cultured tumor cells in the logarithmic phase were treated with 0.25% trypsin (Gibco; Thermo Fisher Scientific, Inc.), and suspended in complete medium with 10% fetal bovine serum (FBS), 80 U/ml penicillin, and 100 µg/ml streptomycin (Gibco; Thermo Fisher Scientific, Inc.). The cells were counted, and 50, 100, and 200 cells/well were seeded in 6-well plates; the plates were incubated at 37°C in a humidified atmosphere containing 5% CO_2_ for 2 weeks. When macroscopic cell colonies were visible in the plates, the supernatant was discarded; the cells were washed three times with cold phosphate buffered saline (PBS), fixed with absolute methanol for 20 mins, and stained with 0.1% crystal violet for 30 mins at room temperature. Subsequently, the cells were washed with PBS and air-dried. The number of cell colonies was counted visually using a microscope; cell clusters containing >50 cells were counted as a single colony.

**Wound-scratch assay, cell migration and invasion assay**

Wound-scratch assay was performed to evaluate the migration capability of control and treated cells. Cells were plated in six-well plates and cultured at 37°C with 5% CO_2_; wound tracks were created by scraping the cell monolayer with sterile pipette tips. Detached cells were removed by washing with PBS and the cells in the plates were subsequently incubated in complete medium. The ability of cells to migrate to the wound areas was assessed by comparing the wound tracks in the images taken after 0 and 26 h. Using Image-J software, the migration index was calculated using the following formula: [(wound area at 0 h) − (wound area at indicated time point)]/ (wound area at 0 h). A higher score indicated better migration ability.

Migration and invasion ability were also measured via transwell migration assay using cell culture inserts (8 μm; Corning Inc., Corning, NY) placed in a 24-well plate. Cells (5×10^4^ cells per insert) in 200 μL medium without FBS were added to the upper chamber. At the same time, culture medium containing 20% FBS was added to the lower chamber (coated with matrigel), after which the plates were incubated for 12 h at 37°C. After removing the medium without FBS and the transwell inserts, the cells in the lower chamber were fixed in methanol for 30 min and stained with 0.1% crystal violet for 30 min. Migration and invasion ability was assessed by counting the number of cells per field. Photos were taken at 40 × magnification, and cells were counted from at least 5 different fields. Three independent experiments were performed.

**Supplementary Figures**


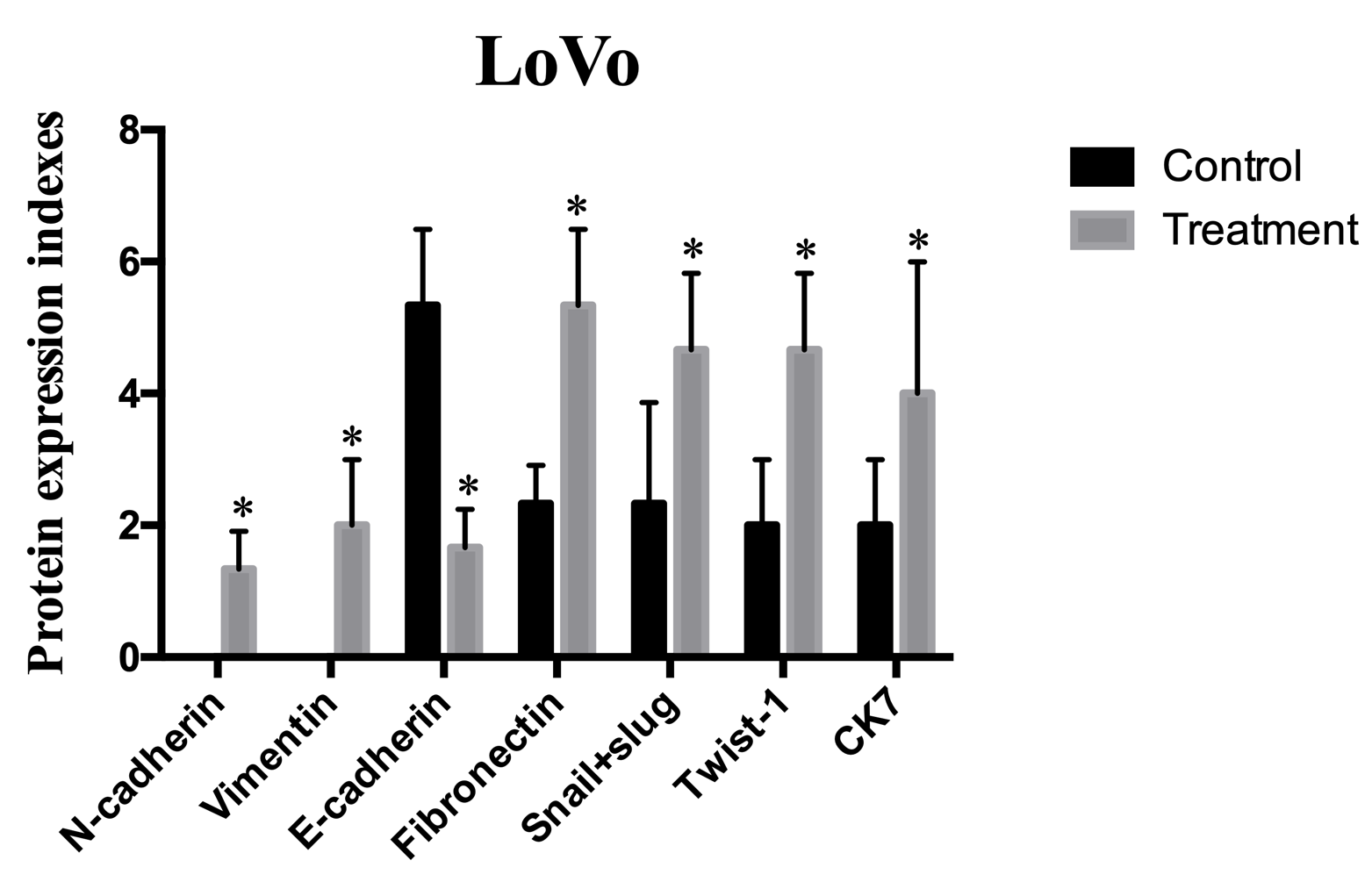


Supplementary Figure S1. Quantitative results of the expression differences of N-cadherin, Vimentin, E-cadherin, Fibronectin, Snail+Slug, Twist-1, and CK7 in LoVo control cells and treatment cells are shown as histograms. Each bar represents the mean ± SD of three independent experiments (**P*< 0.05).


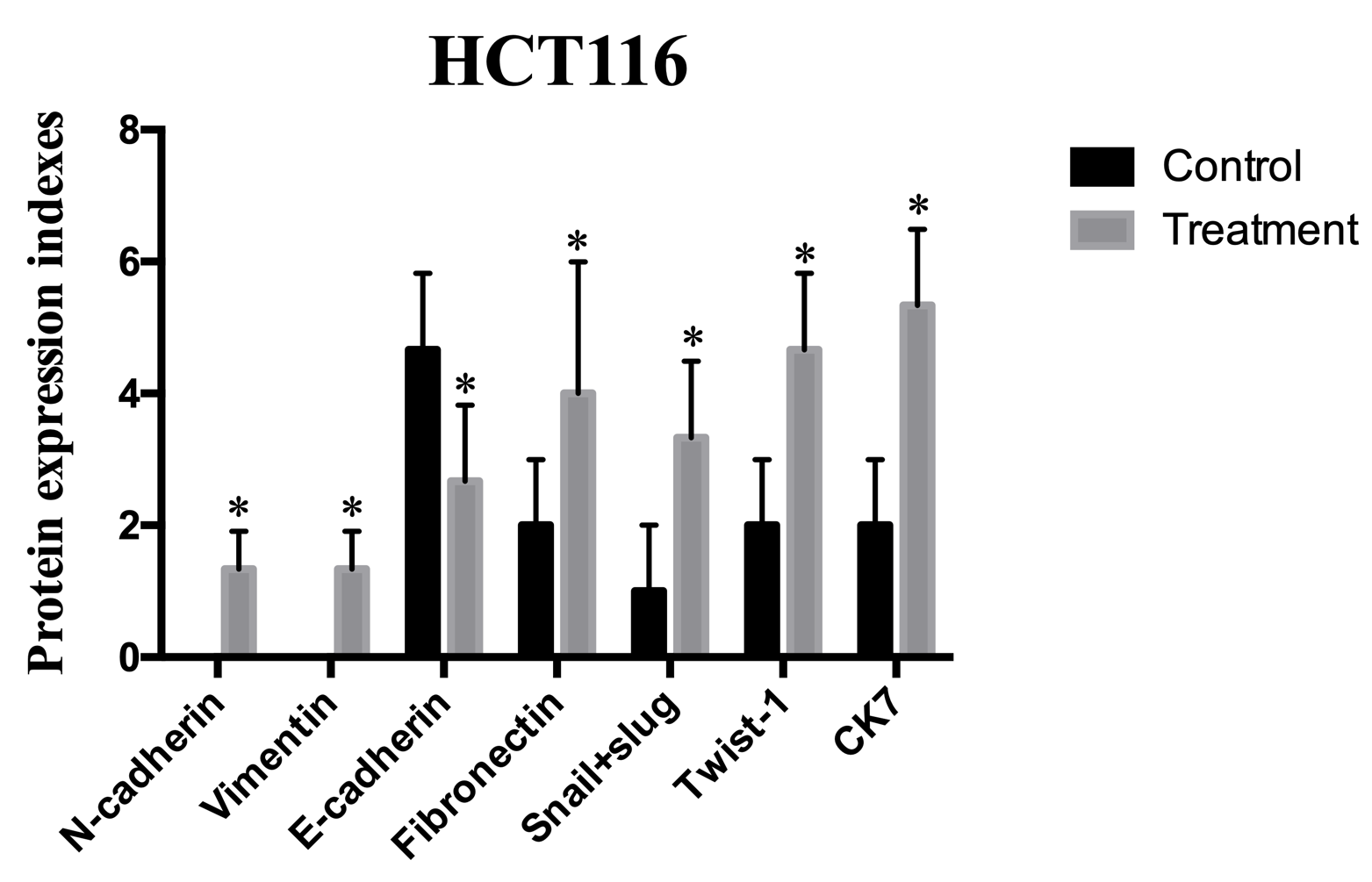


Supplementary Figure S2. Quantitative results of the expression differences of N-cadherin, Vimentin, E-cadherin, Fibronectin, Snail+Slug, Twist-1, and CK7 in HCT116 control cells and treatment cells are shown as histograms. Each bar represents the mean ± SD of three independent experiments (**P*< 0.05).

**Supplementary Tables**

Supplementary Table S1. Different concentrations and durations of chemotherapeutic treatment in different cell lines

| **Cell lines** | **Treatments** | **Concentration** | **Duration** |
| --- | --- | --- | --- |
| LoVo | 5-fluorouracil | 150 µM | 48 hours |
|  | Oxaliplatin | 2 µM | 48 hours |
|  | irinotecan | 200 µM | 48 hours |
| HCT116 | capecitabine, | 200 µM | 48 hours |
|  | oxaliplatin and | 5 µM | 48 hours |
|  | irinotecan | 200 µM | 48 hours |

Supplementary Table S2. Detail information of antibodies used in this study.

| **Antibody** | **Company (catalog number)** | **Dilution** |
| --- | --- | --- |
| CK7 | MXB Biotechnologies Inc., Fuzhou, China, Kit-0021 | 1:300 (WB); 1:100(ICC) |
| Slug | Santa Cruz Bitotechnology, INC (sc- 15391) | 1:300 (WB); 1:100(ICC) |
| Snail | Novus (NBP1-80022) | 1:1000 (WB); 1:100(ICC) |
| Vimentin | Abcam (EPR3776) | 1:100 (ICC); 1:100(ICC) |
| E-cadherin | Abcam(ab7047) | 1:100 (ICC); 1:100(ICC) |
| Twist | Santa Cruz Bitotechnology, INC (sc- 6269) | 1:300 (WB); 1:100(ICC) |
| Fibronectin | BD Pharmingen (no. 61007) | 1:5000(WB); 1:500(ICC) |
| N-cadherin | Cell Signaling (13116) | 1:100 (ICC) |
| β-actin | Sigma-Aldrich (A5316) | 1:100000 (WB) |

ICC: immunocytochemical staining; WB: western blot.
